# Correlation analysis between foveal avascular zone and near peripheral retinal hypoperfusion in multiple sclerosis: a wide field optical coherence tomography angiography study

**DOI:** 10.3389/fmed.2022.1032514

**Published:** 2022-10-24

**Authors:** Daniela Montorio, Gilda Cennamo, Antonio Carotenuto, Maria Petracca, Vincenzo Brescia Morra, Ciro Costagliola

**Affiliations:** ^1^Eye Clinic, Department of Neurosciences, Reproductive and Odontostomatological Sciences, University of Naples Federico II, Naples, Italy; ^2^Eye Clinic, Department of Public Health, University of Naples Federico II, Naples, Italy; ^3^Department of Neurosciences, Reproductive and Odontostomatological Sciences, University of Naples Federico II, Naples, Italy

**Keywords:** widefield OCTA, solix OCT, RRMS, FAZ, vessel density, multiple sclerosis

## Abstract

The identification of non-invasive biomarkers to investigate and monitor retinal structural and vascular changes in multiple sclerosis (MS) represents an interesting source of debate. Until now optical coherence tomography angiography (OCTA) evaluated the foveal avascular zone (FAZ) and areas of retinal non-perfusion only in the macular region in MS patients. It could be interesting to identify possible biomarkers, useful in assessing the ischemic areas also in the near peripheral retina, since FAZ enlargement and the areas of peripheral retinal non-perfusions share common pathogenic processes. In this cross-sectional study, we investigated the correlation between the FAZ area and retinal vessel density (VD) in the near peripheral retina by new wide-field optical coherence tomography angiography (OCTA) in patients affected by relapsing-remitting multiple sclerosis (RR-MS). Moreover, we compared the FAZ area and the VD of superficial and deep capillary plexuses in the fovea region and in the near peripheral retina (6.4 × 6.4 mm) between RR-MS patients and healthy controls by means of a Solix full-range OCTA. Last, we also detected the changes in structural OCT parameters (ganglion cell complex and retinal nerve fiber layer). Thirty-three eyes of 33 RR-MS patients and 35 eyes of 35 healthy controls were enrolled. RR-MS patients showed a lower VD in the superficial capillary plexus and a significant increase in the FAZ area compared with controls. The deep capillary plexus revealed a reduced VD although not statistically significant in patients with respect to controls. In the patients' group, the FAZ area showed significantly negative correlations with VD of superficial capillary plexuses in the foveal and whole region, while the FAZ area did not negatively correlate with the VD of the deep capillary plexus. The significant correlations among OCTA parameters could demonstrate the FAZ area as a possible biomarker for assessing the perfusion status in the near peripheral retina, useful in RR-MS management. These findings could confirm the role of vascular dysfunction in the pathogenetic mechanisms of MS.

## Introduction

Multiple sclerosis (MS) is an autoimmune, inflammatory, and degenerative central nervous system disorder (1); however, changes in the cerebral vasculature can occur and contribute to MS pathophysiology (2). Understanding the cerebral vascular changes in MS could potentially lead to better management of patients and improved diagnosis and prevention of disease progression.

Therefore, the identification of biomarkers for MS represents a crucial challenge for researchers.

Due to the close anatomical relationship between the brain and ocular vasculature, the retinal perfusion impairment in MS patients, demonstrated in several reports, was suggested as possibly having a crucial role in cerebral hypoperfusion in MS pathogenesis (3–5).

The introduction of optical coherence tomography angiography (OCTA) provided a noninvasive imaging technique to perform qualitative and quantitative analyses of the retinal vascular status in MS patients in different disease stages or in longitudinal studies (6–12).

Previous studies, using OCTA, found a significant impairment of retinal blood flow focusing on limited retinal fields, namely the macular region or the foveal avascular zone (FAZ) (13–17).

The FAZ area, localized inside the macula, is surrounded by interconnected fine capillaries at the margin of the fovea coming from superficial and deep retinal vascular networks. This area is extremely vulnerable to hypoperfusion damages that cause the drop out of these terminal capillaries resulting in an enlargement of the FAZ (18).

Therefore, the changes in the FAZ area reflect the impairment of retinal vascular networks and it represents a sensitive biomarker for ischemic processes that occur in retinal vasculopathy, such as in diabetic retinopathy and in retinal vascular occlusions (19, 20). In these diseases, the visualization of larger retinal areas by means of OCTA was helpful to assess the areas of non-perfusion and microvascular abnormalities (21, 22).

Since both FAZ enlargement and retinal peripheral ischemia share a common pathogenic mechanism that involves a capillary non-perfusion, it could be useful to investigate the relationship between retinal peripheral ischemia and macular ischemia in MS eyes by evaluating the retinal vessel density (VD) and FAZ area, respectively (23).

The new OCTA algorithms, recently developed, allowed for the objective assessment of the retinal VD by advanced post-processing imaging measurements in larger retinal areas involving the near peripheral retina (24). The study of these areas could be useful in confirming the possible implication of vasculature impairment as a crucial event in MS pathogenesis.

The purpose of this study was to evaluate in MS patients the retinal vascular perfusion in the near peripheral retina and its relationship with the FAZ area, by OCTA. The FAZ could represent a valid biomarker for vascular status in the near peripheral retina.

## Methods

### Subjects

In this cross-sectional study, we enrolled consecutive 33 relapsing-remitting multiple sclerosis (RR-MS) patients at the “Federico II” University of Naples MS Centre, from December 2021 to February 2022.

The patients met RR-MS diagnosis according to the 2017 McDonald criteria (25). Family history, motor disability assessed through the Expanded Disability Status Scale (EDSS), disease duration, and previous relapses were recorded for all patients.

Exclusion criteria were the presence of systemic diseases that may influence the retinal blood flow such as vascular comorbidities (hypertension, diabetes, and heart diseases), chronic obstructive pulmonary disease, or habits such as smoking. We excluded clinically relevant lens opacities, low-quality images obtained with Spectral Domain (SD)-OCT and OCT-A, myopia greater than six diopters, history of intraocular surgery, vitreoretinal and retinal vascular diseases, glaucomatous neuropathy, uveitis, and congenital eye disorders.

We excluded patients with previous relapses and any history of optic neuritis, in order to avoid a bias related to optic nerve direct damage.

Last, we enrolled 35 healthy controls that presented normal neurological and ophthalmic examinations.

All subjects underwent a complete neurological and ophthalmological examination including the evaluation of best-corrected visual acuity, slit-lamp biomicroscopy, fundus examination, axial length (AL) measurements with IOL Master (26, 27), and SD-OCT and OCTA. Two masked and independent observers (GC; DM) carefully reviewed the SD-OCT and OCTA images.

The study was approved by the Institutional Review Board of the University of Naples “Federico II” and all investigations adhered to the tenets of the Declaration of Helsinki (protocol number: 142/19). Written informed consent was obtained from each of the subjects enrolled in the study.

### Optical coherence tomography and optical coherence tomography angiography

All images were acquired with a Solix full-range OCT (Optovue Inc., Freemont, CA, USA), a new ultra-high-speed spectral domain device that operates at 120,000 A-scans per second with the split spectrum amplitude-decorrelation angiography (SSADA) algorithm (28).

Before imaging acquisition, each eye was dilated by instilling 1% tropicamide eye drops. The participants underwent scanning protocols for structural OCT images consisting of the evaluation of the circumpapillary retinal nerve fiber layer (RNFL), analyzing the optic nerve head map protocol using a 3.45 mm radius ring centered on the optic disc. The ganglion cell complex (GCC) thickness was analyzed by scans centered 1 mm temporal to the fovea over a 7 × 7 mm^2^ area in the macular region.

Each OCT scan was evaluated according to APOSTEL recommendations and the OSCAR-IB protocol for quality control (29, 30).

Low-quality scans (i.e., eye blinking or scan with significant motion artifacts) and signal strength index (SSI) <8/10 were excluded from the analysis.

OCTA images were collected by the AngioVue software that automatically evaluated the VD of the superficial and deep capillary plexuses (SCP, DCP) in the whole region that corresponds to a 6.4 × 6.4 mm field centered on the fovea, namely in the near peripheral retina.

Inside the whole region, the scans also analyzed the fovea region that corresponds to the center ring (the area within the central 1-mm ring of the ETDRS grid) (31). In the foveal region, AngioVue software automatically calculated the FAZ area in square millimeters over the 6.4 × 6.4 mm area in the full retinal plexus.

Each OCTA image underwent Motion Correction Technology and 3D projection artifact removal to improve image quality. Low-quality scans and SSI <8/10 were rejected.

### Statistical analysis

Statistical analysis was performed with the Statistical Package for Social Sciences (Version 25 for Windows; SPSS Inc, Chicago, Ill, USA). Normality distribution was assessed through a Shapiro–Wilk test and model residuals were visually inspected to ensure model homoscedasticity. We explored differences in VD of superficial, deep vascular networks, and the FAZ area, as well as structural OCT parameters (GCC average and RNFL average) through general linear models, including age and sex as covariates and as a group as a factor of interest. Correlations among OCTA parameters in each study group were assessed using Pearson's correlation coefficients.

The agreement between two observers in the measurement of SD-OCT and OCTA parameters was assessed using the intraclass correlation coefficient. A *p-*value < 0.05 was considered statistically significant.

## Results

### Demographic and clinical features

Thirty-three RR-MS patients for a total of 33 eyes (17 women, 16 men; mean age 38.2 ± 6.2 years) and 35 HCs for a total of 35 eyes (19 women, 16 men; mean age 38.1 ± 7.3 years), were enrolled. There were no significant differences for age, sex, or best-corrected visual acuity between the two groups.

The mean AL of the RR-MS patients was 23.7 ± 1.4 mm (range: 21.5–26.8 mm) and the mean AL of the HCs was 23.5 ± 1.0 mm (range: 21.2–26.4 mm), with no statistically significant difference was found between the two groups (*p* = 0.24).

### SD-OCT and OCTA

**Figure 1:**
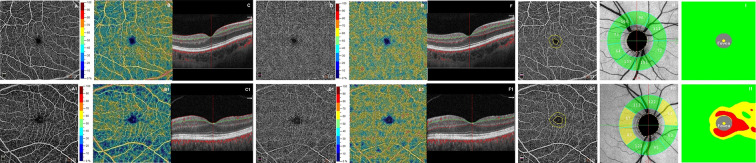
First row shows optical coherence tomography angiography (OCTA) images from a healthy subject's right eye (male, 30 years): normal superficial and deep capillary plexuses in terms of retinal vasculature texture **(A,D)** and in vessel density **(B,E)**. The OCT B-scan **(C,F)** shows normal retinal profile, thickness. The foveal avascular zone (FAZ) shows a normal size of the area **(G)**. Structural OCT reveals normal thicknesses of retinal nerve fiber layer (RNFL) **(H)** and ganglion cell complex (GCC) **(I)**. The second row shows a patient's right eye (female, 31 years) affected by relapsing-remitting multiple sclerosis. OCTA images show in superficial capillary plexus a rarefaction in vascular texture **(A1)** and in vessel density **(B1)**. The deep capillary plexus reveals focal rarefactions in terms of retinal vasculature texture **(D1)** and in vessel density **(E1)**. The OCT B-scan **(C1, F1)** shows a normal retinal profile and thickness. The foveal avascular zone (FAZ) shows an evident enlargement of the area **(G1)**. Structural OCT reveals several zones of reduced thicknesses of RNFL and GCC respect to healthy subject **(H1, I1)**.

In the SD-OCT exam, RR-MS patients showed a significant thinning of GCC and RNFL with respect to the healthy controls (coeff. β = −10.235; *p* = 0.026; coeff. β = −9.768, *p* = 0.031). The VD of the superficial capillary plexus in the whole and foveal regions was significantly lower in the RR-MS group compared with the healthy controls (coeff. β = −4.584; *p* = 0.021; coeff. β = −4.736; *p* = 0.032). Whereas the deep capillary plexus showed a reduced VD, although not statistically significant, in patients with respect to controls. The FAZ area was significantly increased in patients with respect to controls (coeff. β = 0.117; *p* = 0.003) (Table 1, Figure 1).

**Table 1:** Demographic, clinical features and differences in OCT/OCTA parameters between RRMS patients and healthy controls.

	**Control**	**RR-MS**	**β**	***p*** **-value**
**Eyes (N)**	35	33	–	–
**Age (years)**	38.1 ± 7.3	38.2 ± 6.2	–	0.874
**Sex (female/male)**	19/16	17/16	–	0.732
**EDSS**	–	2.7 ± 1.03	–	–
**Disease duration (years)**	–	5 ± 2.4	–	–
**OCT-A parameters (%)**				
SCP Whole	51.22 ± 3.72	46.61 ± 4.45	−4.584	0.021*
SCP Fovea	35.37 ± 5.96	31.14 ± 4.52	−4.736	0.032*
DCP Whole	55.18 ± 4.82	53.21 ± 5.02	−1.527	0.146
DCP Fovea	31.41 ± 3.22	30.11 ± 3.86	−1.612	0.257
FAZ	0.201 ± 0.12	0.319 ± 0.19	0.117	0.003*
**OCT parameters (μm)**				
GCC average	100.6 ± 6.25	90.31 ± 7.52	−10.235	0.026*
RNFL average	103.4 ± 7.12	94.27 ± 7.41	−9.768	0.031*
**Best-corrected visual acuity (logMAR)**	0.01 ± 0.03	0.02 ± 0.04	0.034	0.826

In the RR-MS group, the FAZ area disclosed significant negative correlations with VD of the superficial capillary plexus in the fovea region (*r* = −0.68, *p* = 0.015), as well as, in the whole region (*r* = −0.76, *p* = 0.006). Likewise, the FAZ area did not significantly correlate with the VD of the deep capillary plexus in either region. The relationship between SD-OCT and OCTA parameters showed a statistically significant correlation only between the GCC thickness and VD of the superficial capillary plexus (*r* = 0.71, *p* = 0.024). Last, no significant correlation was found between MS severity and duration, and OCTA parameters.

In the analysis of healthy controls, there were no statistically significant correlations between the FAZ area and both retinal vascular networks in the fovea and near peripheral retina. The agreement between the two observers for measuring the SD-OCT and OCTA parameters was excellent, with an intraclass correlation coefficient of 0.93.

## Discussion

This is the first study to describe the retinal VD in the near peripheral retina by means of OCTA in RR-MS patients. In these areas, the VD of SCP was significantly reduced with respect to controls, and the FAZ area was significantly greater with respect to controls. Also, the structural SD-OCT showed a significant thinning of GCC and RNFL in MS patients. Thanks to this new OCTA algorithm, the Solix Full Range OCT, the ultra-high-speed scans produce a large field of view and outstanding resolution imaging and this allows, for the first time, a deep and wide study of the retinal vasculature.

The findings of this study revealed a diffuse status of retinal hypoperfusion that affected wider retinal areas, beyond the macular region reaching the near peripheral retina where the density of ganglion cells is reduced (31). The significant correlation between the reduced VD of superficial capillary plexus in the whole retina area and the thinning GCC would confirm the significant role of the vascular factor in pathogenetic processes involved in MS. Indeed, even in absence of ongoing inflammatory events, such as optic neuritis, the retinal vascular damage is accompanied by the loss of structural OCT parameters which could demonstrate a progressive relapse-independent neurodegenerative disease activity.

Few studies focused on the changes in the FAZ area in MS (15–17) showing ambiguous results, probably due to differences in OCTA devices (32–34). Only one study, conducted by Hayati et al., reported a significant correlation only between the FAZ area and the inner rings of SCP, analyzing a 3 × 3mm macula area (16).

In this study, we obtained quantitative and precise information, using advanced OCTA software, about the enlargement of the FAZ area that may reflect an ischemic process. Until now, previous research demonstrated the increased FAZ area associated with the severity of diabetic retinopathy and retinal vein occlusion, demonstrating the sensible role of the FAZ in vascular damage (21, 35). The novelty of this study is that it is the first to show the significant relationship between the larger FAZ area and loss of VD in SCP in the whole region, suggesting that the FAZ area may help to assess hypoperfusion in the near peripheral retina in MS patients. This association would support the hypothesis that both of these conditions share a common pathogenic mechanism represented by diffuse microvascular damage that could represent a reflection of the global brain hypoperfusion (36). Indeed, perfusion studies based on multiple imaging modalities reported a reduced cerebral blood flow in both the brain's gray and white matter in MS, highlighting hypoxia as a crucial key in neuronal loss in MS (37–40).

The present study has several limitations including the small sample size and the possible influence of immunotherapy on retinal vascularization. It is an exploratory study that in the future needs to enroll a greater number of cases and follow the vascular changes in the peripheral retina in different MS stages and during disease activity. Despite these limitations, this study presents several strengths including the quantitative and objective assessment of OCTA parameters, the use of masked and independent observers for OCT and OCTA parameters, and rigorous exclusion criteria (e.g., optic neuritis that could affect the retinal VD).

In conclusion, this study demonstrated that the process of hypoperfusion in MS represents a crucial event, capable of involving wide retinal areas, supporting the hypothesis of vascular dysfunction in the pathogenetic mechanisms of MS. Moreover, these interesting findings provided a new insight into the FAZ area that could be used as a possible biomarker for evaluating the perfusion status in the near peripheral retina in MS patients.

## Data availability statement

The raw data supporting the conclusions of this article will be made available by the authors, without undue reservation.

## Ethics statement

The studies involving human participants were reviewed and approved by the Institutional Review Board of the University of Naples Federico II. The patients/participants provided their written informed consent to participate in this study.

## Author contributions

GC and VB conceived and designed the study. DM performed data collection, analyzed data, and designed the statistical analysis. DM, GC, AC, and MP wrote the manuscript. DM and GC revised the manuscript. GC, VB, and CC undertook supervision. All authors contributed to the article and approved the submitted version.

## Conflict of interest

The authors declare that the research was conducted in the absence of any commercial or financial relationships that could be construed as a potential conflict of interest.

## Publisher's note

All claims expressed in this article are solely those of the authors and do not necessarily represent those of their affiliated organizations, or those of the publisher, the editors and the reviewers. Any product that may be evaluated in this article, or claim that may be made by its manufacturer, is not guaranteed or endorsed by the publisher.
